# Future Trends in Asymmetric Organo-Metal Combined Catalysis

**DOI:** 10.1021/acscentsci.5c00393

**Published:** 2025-07-04

**Authors:** Dian-Feng Chen, Jin Song, Liu-Zhu Gong

**Affiliations:** † Hefei National Research Center of Physical Sciences at the Microscale and Department of Chemistry, 12652 University of Science and Technology of China, Hefei 230026, China; ‡ Institutes of Physical Science and Information Technology, 12487 Anhui University, Hefei 230601, China; § School of Materials and Chemistry, Southwest University of Science and Technology, Mianyang 621010, China

## Abstract

Asymmetric organo-metal combined catalysis, which integrates the catalytic functions of chiral organocatalysts and metal complexes, enables the enantioselective formation of challenging chemical bonds and facilitates cascade transformations, often without the need for intermediate purification. Since its inception in 2001, this paradigm has evolved into a versatile strategy for the rapid construction of molecular complexity with a high level of enantioselectivity. In this Outlook, we have highlighted the most recent contributions to this field, showcasing exciting opportunities to overcome current efficiency limits. Looking ahead, we foresee the continued evolution of asymmetric organo-metal catalysis, particularly through the exploration of new catalyst scaffolds, the incorporation of external stimuli, the use of heterogeneous metal catalysts, and the application in macromolecular synthesis.

## Graphical abstract

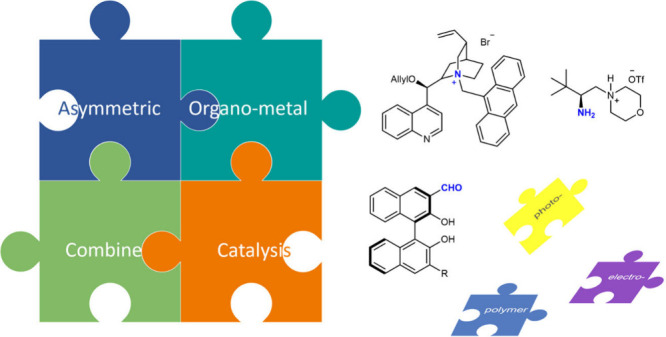

## 1 Introduction

Collaboration between catalyst entities is prevalent in nature, as exemplified by the biosynthesis of structurally complicated big molecules from fundamental small compounds based on reaction sequences enabled by multiple catalysis. Notably, such a cooperative or relay catalysis might even happen in prebiotic metabolism.^1^ A similar concept was applied to asymmetric catalysis two decades ago. In 2001, Gong and Mi^2^ demonstrated that chiral phase-transfer catalysts (PTCs) could cooperate with an achiral palladium complex to enable asymmetric allylic alkylation reactions (AAAs). Through deprotonation of *tert*-butyl­(diphenylmethylene)­glycinate **1** by CsOH and subsequent ion exchange with a cinchonidine-derived chiral ammonium salt, a chiral cation-paired nucleophile **INT-1** was formed, while the oxidative addition of allyl acetate **2** to the palladium complex gave an electrophilic π-allylpalladium species **INT-2** (Scheme 1). The merger of these intermediates successfully produced α-alkylated glycinate **3** with a moderate enantiomeric excess (*ee*) of 59%. At the same time, Takemoto et al.^3^ independently reported a similar catalytic system combining a chiral ammonium salt **5** and a palladium-triphenylphosphite complex, which enhanced the enantioselectivity to 94% *ee* in the same reaction.

**Scheme 1:**
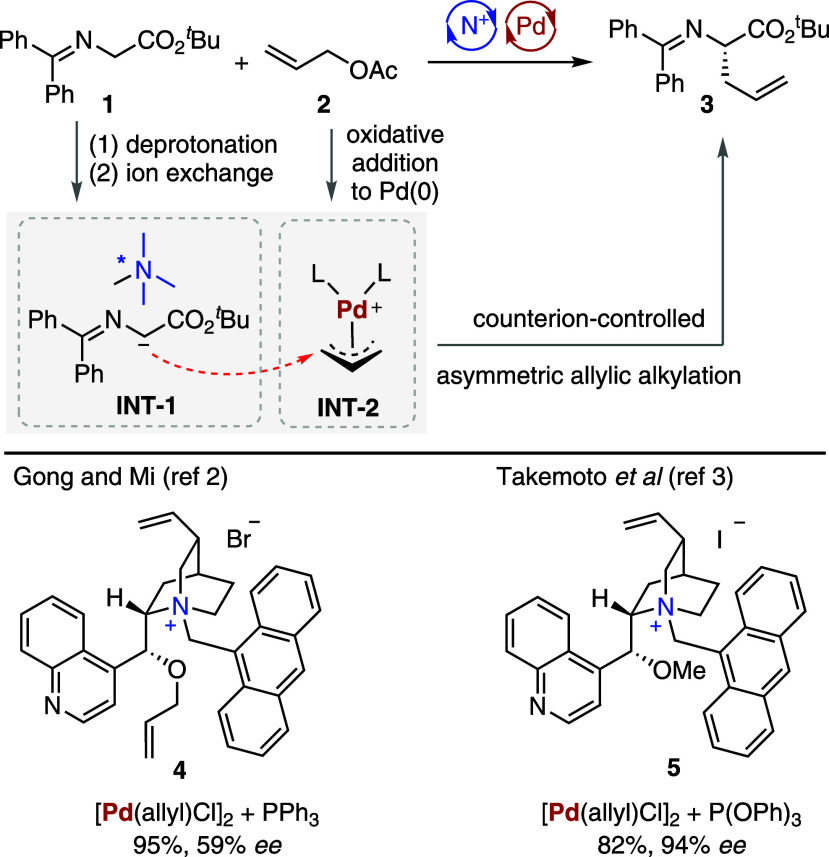
Chiral Ammonium Salts and Achiral Palladium Cooperative Catalysis

Since these inaugural findings,^2,3^ the combination of chiral PTCs, such as chiral ammonium- and phosphonium salts, with achiral transition metal complexes has evolved as a new platform for AAA reactions.^4,5^ A broad range of soft nucleophiles including α-imino esters,^6–8^ oxindoles,^9,10^ and α-cyanoesters^11^ have been employed to couple with electrophilic π-allyl-palladium species that are in situ generated from allyl acetates,^6,7^ β,γ-unsaturated ketones,^9^ and 1,3-dienes,^10^ or with π-allyl-iridium species to tune the linear/branch selectivity.^8^ Notably, Chen and co-workers demonstrated the use of a cinconine-derived ammonium salt to stabilize π-allyl-palladium-based 1,4-carbodipoles by forming a compact diploid ion-pair species.^12^ This distinct double activation strategy has enabled a highly regio-, diastereo-, and enantioselective [4 + 2] annulation reaction with 2-alkylidene indene-1,3-diones.

Chiral PTC-Pd or Ir-cocatalyzed AAA reactions have underlined the crucial role of ion-pairing interactions in the enantio-differentiating step. This mode then evolves from the chiral cation-nucleophile attraction into an unprecedented chiral cation-transition metal complex, significantly expanding the chemical space of chiral PTC-transition metal cooperative catalysis.^13,14^ The metal-involved chiral cation-pairing can date back to a KMnO_4_-mediated oxidative cyclization^15^ and dihydroxylation reaction,^16^ in which a hydrogenated cinchonidine-derived PTC **6** provides a chiral cation that stabilizes the resultant organo-permanganate intermediate, leading to moderate enantioselectivity (Figure 1a, left). In 2015, Tan and co-workers reported an even more effective bisguanidinium chloride catalyst for KMnO_4_-mediated dihydroxylation and oxohydroxylation of acrylate derivatives.^17^ They further developed a dual tungstate/bisguanidinium chloride system, which assembled into chiral cation-directed tungstate catalyst **7** (Figure 1a, center). This system has rendered highly enantioselective sulfoxidation of thioethers^18^ and epoxidation of alkenyl amides.^19^ In a related approach, Maruoka and co-workers demonstrated that a biaryl-derived spiro PTC **8** (Figure 1a, right) could act as the cation of an alkynyl silver halide anionic species, achieving highly enantioselective alkynylation of isatins.^20^ In an electron-inverse sense, Toste et al. combined robust chiral anionic PTC with palladium catalysis,^21^ opening new avenues for enantioselective difunctionalization of alkenes (Figure 1b), including arylborylation,^22,23^ diarylation,^24^ alkynylborylation,^25^ and other transformations.^26–28^

**Figure 1:**
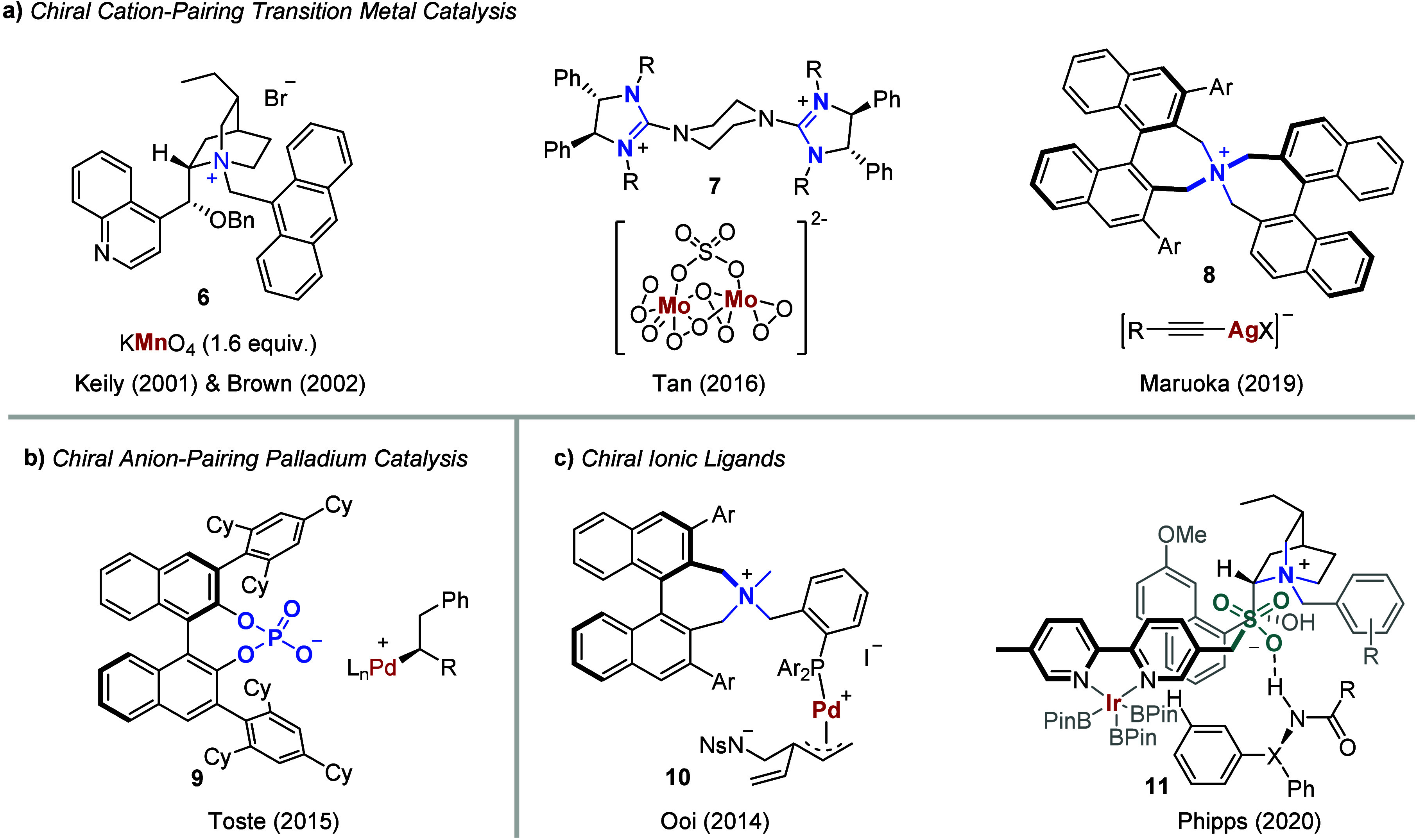
PTC activation of transition metal catalysis: (a) chiral cation-pairing transition-metal catalysis; (b) chiral anion-pairing palladium catalysis; (c) chiral ionic ligands.

A notable front on the broader application of chiral ion-pairing interactions derived from chiral PTC-transition-metal cooperative catalysis is the stunning design of ionic ligands.^29^ Although ionic ligands seem to be out of the scope of previous “organo-transition metal combined catalysis”, they may be considered as specially designed chiral PTCs, which carry pendant phosphine functionalities to impart supramolecular coordination.^30^ A prime example was that Ooi et al. developed an ion-paired ammonium-phosphine ligand bearing a chiral binaphtholate moiety for the palladium-catalyzed AAA reaction of α-nitrocarboxylate with cinnamyl carbonate.^31^ They also designed a well-defined chiral ammonium-phosphine hybrid ligand **10** (Figure 1c, left) for the [3 + 2] annulation reaction of 5-vinyloxazolidinones with activated trisubstituted alkenes.^32^ The diploid electrostatic interactions effectively prevent the unfavorable intramolecular coordination of the sulfonamide anion to the palladium center, thus enhancing both reactivity and enantioselectivity. Afterward, the Phipps group created a preeminent ligand consisting of *N*-substituted dihydroquinine and 5-bipyridylmethyl sulfonate. This ligand has facilitated iridium-catalyzed enantioselective and desymmetrizing *meta*-C–H borylation of benzhydrylamides and diaryl phosphinamides.^33^ Control experiments probing the ligand-cation interactions have revealed that the anionic sulfonate group anchors to the substrates via hydrogen bonding and/or π–π interactions,^34^ resulting in a highly organized complex **11** (Figure 1c, right) that promotes excellent *meta*-selectivity and enantioselectivity.

In parallel with the development of integrated chiral PTCs and transition-metal catalysis, a variety of distinct chiral organocatalysts, in cooperation with versatile transition-metal complexes, have been explored over the past two decades.^35,36^ This organo-metal cooperative catalysis employs sophisticated but synchronous activation modes to address challenges associated with reactivity and selectivity, including chemo-, regio-, diasetereo-, and enantioselectivity, particularly in the redistribution of chemical bonds to build up molecular complexity. As illustrated in Figure 2, three primary activation models broadly dominate the selection of chiral organocatalysts: iminium/enamine activation with chiral amines (Figure 2a),^37–40^ hydrogen bonding interactions with chiral Brønsted acids (Figure 2b),^41–44^ and nucleophilic activation with chiral Lewis bases, such as nitrogen-heterocyclic carbenes (NHCs),^45^ isothioureas (ITUs)^46,47^ and phosphines^48^ (Figure 2c). The most frequently used organometallic intermediates in these systems include metal π-alkynes,^49^ π-allyl- ^50,51^ and benzyl-metallic species,^52^ metal-complexed allenylidenes,^53^ and acyl metalloids^54^ (Figure 2c). Notably, matching a chiral organocatalyst and a chiral transition-metal complex has enabled extraordinary access to all stereoisomers of products bearing multiple stereogenic centers,^55–57^ including chiral amine/iridium-chiral phosphoramidite-catalyzed α-allylation of aldehydes,^58–62^ chiral ITU/iridium- ^63,64^ or palladium-catalyzed α-allylation of esters,^65^ chiral amine/rhodium-catalyzed hydroalkylation of alkynes,^66^ chiral amine,^67^ or chiral PTC^68^/palladium-catalyzed hydroalkylation of 1,3-dienes, chiral squaramide/nickel-catalyzed α-propargylation of oxindoles,^69^ and chiral NHC/copper-catalyzed β-propargylation of enals.^70^ In a word, a number of preceding reviews on asymmetric organo-metal cooperative catalysis have been published,^71–78^ and we encourage readers to consult them for a comprehensive understanding of the discovery, evolution, and principles underlying this research field.

**Figure 2:**
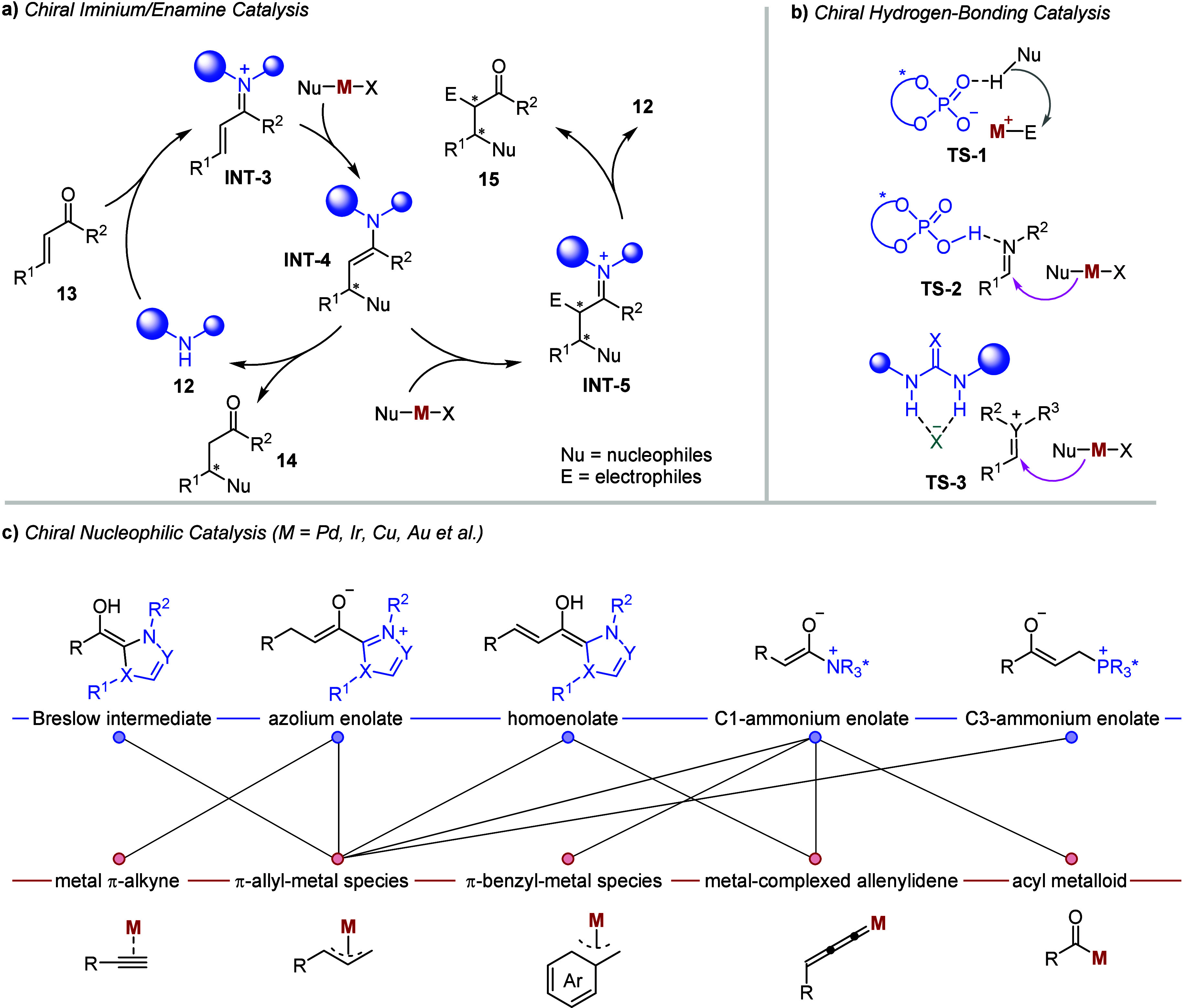
Representative asymmetric organocatalysis that cooperates with metal complexes: a) chiral iminium/enamine catalysis; b) chiral hydrogen-bonding catalysis; c) chiral nucleophilic catalysis.

In this Outlook, we focus on recent advancements in aligning organocatalysts and transition-metal complexes to enhance the synthesis of enantioenriched molecules and explore the future trends in this field. Emphasis has been placed on the rational design of chiral organocatalysts that feature novel scaffolds or new activation modes. We aim to advance the understanding of each catalyst’s role in bond-forming events, thereby unlocking the full potential of this strategy for synthetic chemistry.

This Outlook reviews recent advancements in the alignment of organocatalysts and transition-metal complexes to enhance the synthesis of enantioenriched molecules and explores the future trends in this field.

## 2 New Trends in Organic Synthesis

While systematic combinations of well-established chiral organocatalysts and transition-metal–ligand complexes have led to the early success of asymmetric organo-metal cooperative catalysis, substantial efforts devoted to the development of electronically and sterically distinct catalyst scaffolds are proving essential. These innovations not only enhance bond activation efficacy but also present new paradigms for stereocontrol, thereby fueling the continued growth of this field in addressing the synthetic challenges associated with enantioenriched molecules. In this section, we highlight four key catalyst innovations involving chiral scaffolds that have recently emerged: (1) quaternized cinchona alkaloid cations; (2) chiral aldehyde catalysis; (3) multiple catalyst systems; and (4) external stimuli-endowed organo-metal combined catalysis.

### 2.1 Quaternized Cinchona Alkaloid Cations

Enantioselective C­(sp^3^)–H amination with high regioselectivity and stereoselectivity still remains a grand challenge. In this context, paddlewheel rhodium­(II) dicarboxylate complexes have emerged as efficient catalysts capable of generating rhodium nitrenoids, which can insert into C­(sp^3^)–H bonds to facilitate direct aminative functionalization.^79^ To render an enantioselective C­(sp^3^)–H amination, incorporation of chiral carboxylic acids or carboxamides into the rhodium­(II) complexes remains a unique and effective strategy.^80,81^

The Phipps group has demonstrated an elegant strategy using an anionic sulfonate ligand paired with a quaternized cinchona alkaloid cation, which implements excellent enantiocontrol in an iridium-catalyzed, *meta*-selective arene borylation reaction.^33^ This chiral cation-directed approach was recently extended to the synthesis of a family of ion-paired chiral rhodium­(II) complexes (Scheme 2a).^82^ Compared to classical rhodium dimers bearing chiral carboxylate or carboxamidate ligands, the chiral source of cation-paired rhodium­(II) catalysts, i.e., the quaternized cinchona alkaloid cation, is located at a considerably closer distance from the reactive axial site, which likely influences the enantio-determining step to a higher degree. By using perfluorinated sulfamate ester **20** as the nitrogen source and iodosobenzene (PhIO) as the oxidant, the group evaluated the influence of cinchona alkaloid cation’s absolute configuration on the reaction of 4-phenybutan-1-ol, identifying a catalyst bearing the dihydroquinidine motif (**16**) as particularly effective in creating the optimal chiral environment for this intermolecular benzylic C­(sp^3^)–H amination. Compared to commercially available achiral Rh_2_(esp)_2_, the optimal ion-paired catalyst also resulted in significantly improved yields. This strategy was then applied to a broad scope of 4-arylbutanols **19**, yielding chiral 1,4-aminoalcohols **21** in 29–90% yields and with up to 93% *ee* (Scheme 2b), which is much higher than the *ee* value (74%) achieved by rhodium­(II) catalyst with chiral dicarboxylate.^83^ Notably, removal of the hydroxyl group from the substrate led to a drastically decreased yield and enantioselectivity (∼3% yield, 28% *ee*), while variation in alcohol chain length was found against the best catalyst scaffold for 4-phenybutan-1-ol. These findings highlight the importance of a well-defined chiral pocket formed by the quaternized dihydroquinidine unit, which facilitates superior reactivity and enantio-differentiation through a network of noncovalent interactions between the substrate and catalyst. Subsequently, this benzylic C­(sp^3^)–H amination reaction was expanded to include tertiary amides **22** derived from butyric and valeric acids (Scheme 2c).^84^ More recently, this cinchona alkaloid cation-directed rhodium­(II) catalysis was also applied to an asymmetric nitrene transfer to alkenyl alcohols **24** (Scheme 2d),^85^ providing a unique method for accessing chiral aziridines with varied substitution patterns that have not been covered by traditional rhodium­(II,II) tetracarboxylate dimer catalysts.^86^

**Scheme 2:**
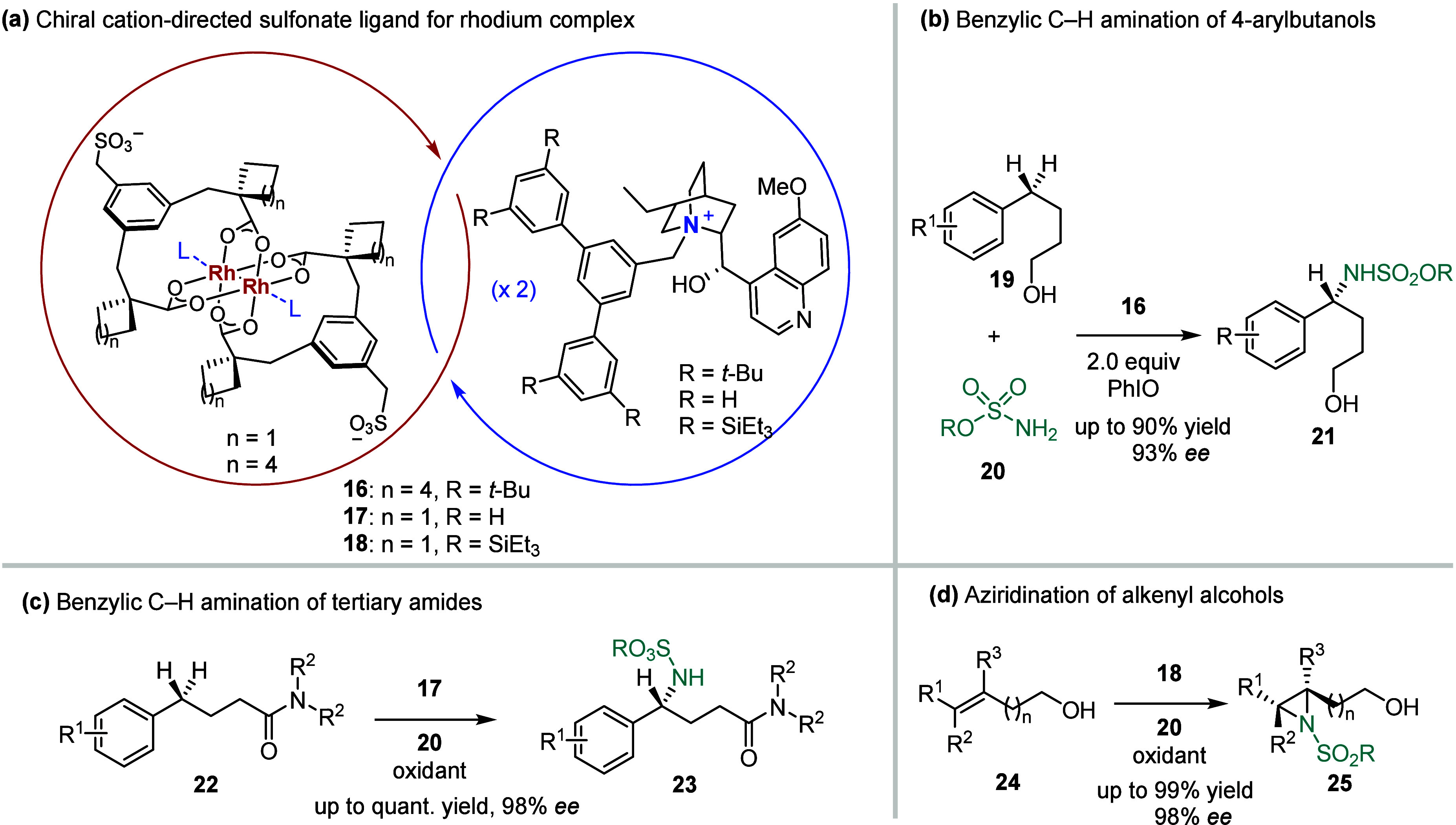
a) Design of Quarternized Cinchona Alkaloid Cation-Directed Sulfonate Ligand for Rhodium Complexes and Their Application in b) Benzylic C–H Amination Reactions of 4-Arylbutanols and c) Tertiary Amides, and in d) Aziridination of Alkenyl Alcohols

### 2.2 Chiral Aldehyde Catalysis

The reversible condensation of chiral amines with aldehydes or ketones forms the fundamental for asymmetric enamine/iminium catalysis.^37–40^ In a reversed sense, catalytic amounts of chiral aldehydes or ketones hold potentials of activating the α-C–H bonds of primary amines by forming imine intermediates.^87–91^ This proposed activation mode mimics the way that pyridoxal-dependent aldolases function in biological systems.^92^ Chiral aldehyde catalysis remained unknown in organic synthetic chemistry until 2011, when Beauchemin and co-workers documented its practicality in an enantioselective Cope-type hydroamination of allylic amines with hydroxyamines.^93^ Since then, Beauchemin,^94,95^ Guo,^96–99^ and Zhao^100–102^ have made significant contributions to advancing this field, achieving a broad range of enantioselective C–C bond-forming transformations (Scheme 3a).

**Scheme 3:**
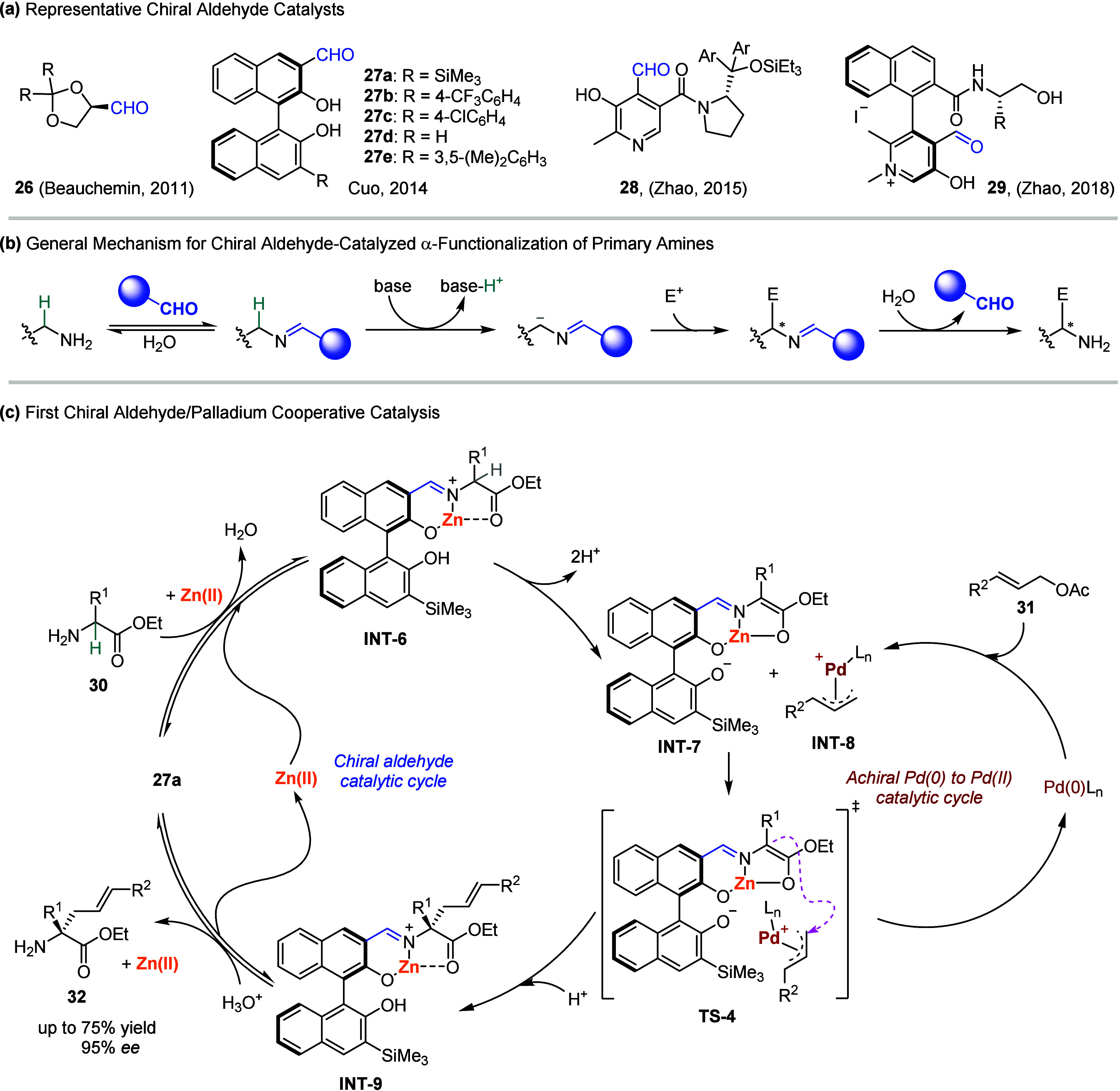
(a) Representative Chiral Aldehydes. (b) Mechanism of Imine Activation of Primary Amines. (c) First Example of Chiral Aldehyde/Palladium Cooperative Catalysis

As illustrated in Scheme 3b, the acidity of α-hydrogen in amines significantly increases upon imine activation by the chiral aldehyde catalyst, which enhances the subsequent reactivity of deprotonation and nucleophilic substitution and addition reactions. Finally, either hydrolysis or amine exchange of the resultant imine intermediate completes the catalytic cycle. Through this mechanism, direct and highly enantioselective α-functionalization of various primary amines has been achieved, including alkylation, arylation, transamination, Mannich reaction, and others.^93–102^

Despite these exciting advancements, the scope of electrophiles has remained narrow, with only highly reactive reagents proving effective. Combining the unique aldehyde chemistry with transition-metal complexes could, in principle, offer a solution to this limitation. In 2019, Guo and co-workers^98^ reported a sophisticated combined catalyst system in which the chiral aldehyde **27a** and ZnCl_2_ imparted double activation of α-imino esters **30** by forming a highly nucleophilic complex, while a Pd(0) complex routinely transformed allyl acetates **31** into the electrophilic π-allyl-palladium species **INT-8**. The coupling of these in situ-generated intermediates afforded optically active α,α-disubstituted α-amino acids **32** in moderate yields and with excellent enantioselectivity (Scheme 3c). Moreover, analogous pathways for generating π-allyl-palladium species from alternative precursors, such as 1,3-disubstituted allylacetates,^103^ aryl iodides/allyl esters or carbonates,^104^ bromomethyl ammonium salt/styrenes,^105^ 1,3-dienes/allenes,^106^ allenylic acetates,^107^ and methylenecyclopropanes,^108^ were also compatible with the chiral aldehyde/Zn­(II)-mediated catalytic cycles.

Developing of catalyst scaffolds with distinct electronic and steric properties can improve bond activation efficacy, offering new paradigms for stereocontrol and fueling the continued growth of asymmetric organo-metal combined catalysis.

In addition to palladium-catalyzed α-allylation reactions, nickel-catalyzed α-propargylation was also successfully integrated with chiral aldehyde catalysis, producing α-propargylamino esters **36** in up to 95% yields and 98% *ee* (Scheme 4a).^109^ Notably, starting from either product (*S*)-**36a** or (*R*)- **36a**, all four stereoisomers of **37** were synthesized with good enantioselectivity, ultimately enabling the stereodivergent total synthesis of natural pyrrolizidine alkaloid NP25302 (Scheme 4b).

**Scheme 4:**
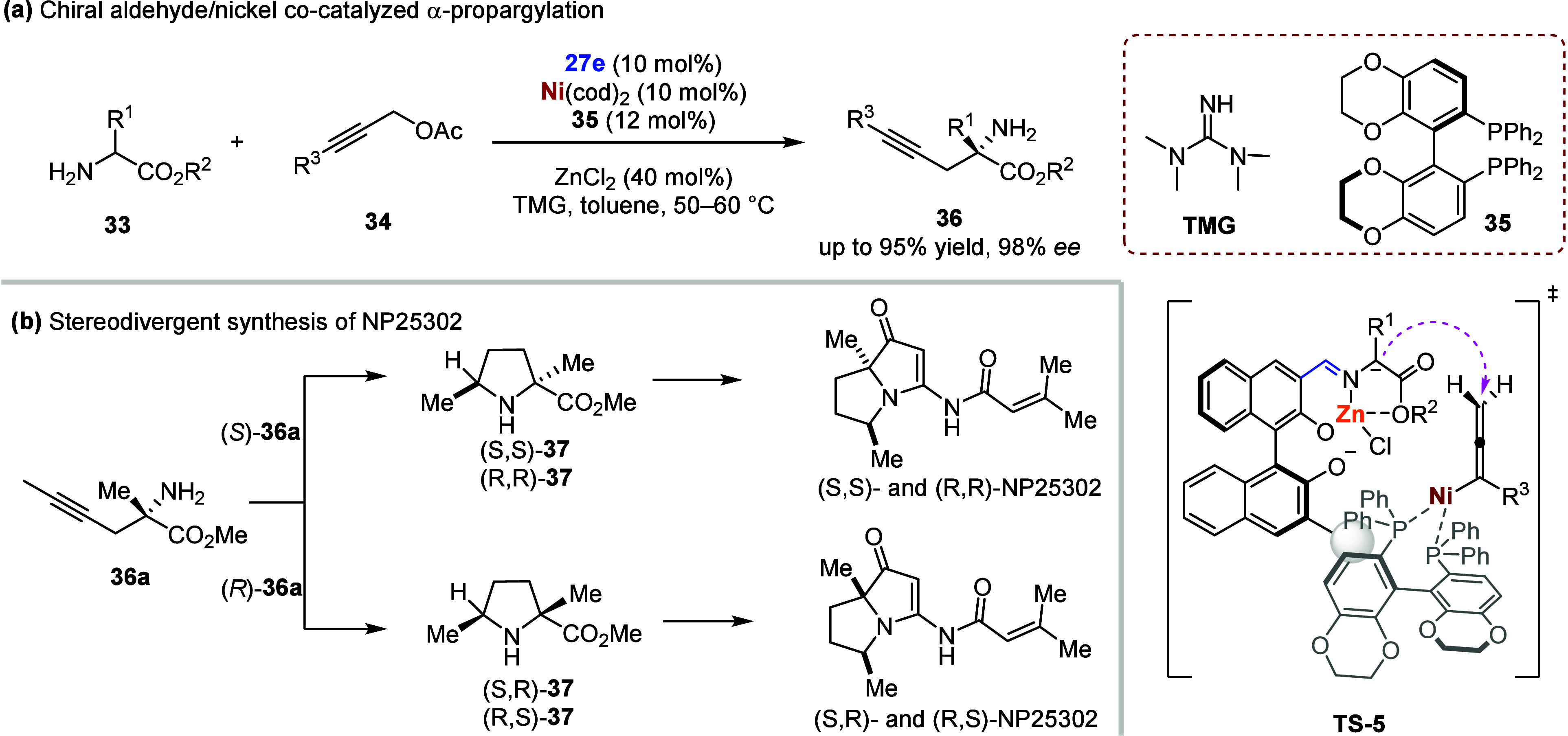
(a) Chiral Aldehyde/Nickel Co-Catalyzed α-Propargylation of Amino Acid Esters. (b) Stereodivergent Synthesis of NP25302

### 2.3 Multiple Catalysis to Streamline Value-Added Chiral Molecule Synthesis

Nature has provided inspiration for improving the overall synthetic efficiency through multiple catalysis. On one hand, multiple-component reactions (MCRs) offer high atom and step economy, enabling expeditious construction of molecules with high structural and functional diversity and intensity. On the other hand, sophisticated multiple catalytic system is capable of forging otherwise inaccessible chemical bonds from readily available feedstocks. These catalysts can operate in either full or relayed cooperation;^110^ the significance of such approaches lies in the in situ manipulation of key yet unstable or transient intermediates.

A highly reactive intermediate recently identified for asymmetric MCRs is the transition metal-associated protic ammonium/onium ylides, generated via the catalytic insertion of carbenoids into heteronucleophiles.^111^ Trapping these active species with electrophiles through delayed proton transfer provides opportunities for the development of novel MCRs. In 2008, Hu and Gong demonstrated that chiral phosphoric acid could impart a high level of enantio-differentiation in the addition of rhodium-associated onium ylides to imines,^112^ laying the foundation for subsequent enantioselective trapping with a range of stable, unsaturated electrophiles. More recently, Xu, Liang, and Hu presented a remarkable ternary cooperative catalytic system consisting of Rh_2_(OAc)_4_, an achiral Pd-complex, and chiral phosphoric acid (CPA) **41**, which extended the trapping process to substitution-type interception (Scheme 5).^113^ Specifically, catalytic N_2_-extrusion of the diazo substrate **38** by Rh_2_(OAc)_4_ produced a highly active rhodium carbenoid **INT-13**, which underwent a O–H insertion reaction with alcohol **39** to give an oxonium ylide **INT-14**. Synchronously, oxidative addition of allyl carbonate **40** to the Pd(0) complex afforded π-allyl-Pd species **INT-11**. The combination of two in situ generated active intermediates—enols **INT-15** derived from the oxonium ylide-**INT-14** and π-allyl-Pd species **INT-11**—resulted in the formation of chiral α,α-disubstituted ketones **42** in high yields and with excellent enantioselectivity. Control experiments and DFT calculations revealed that CPA played a crucial role in enhancing both the reactivity and enantioselectivity via hydrogen bonding.

**Scheme 5:**
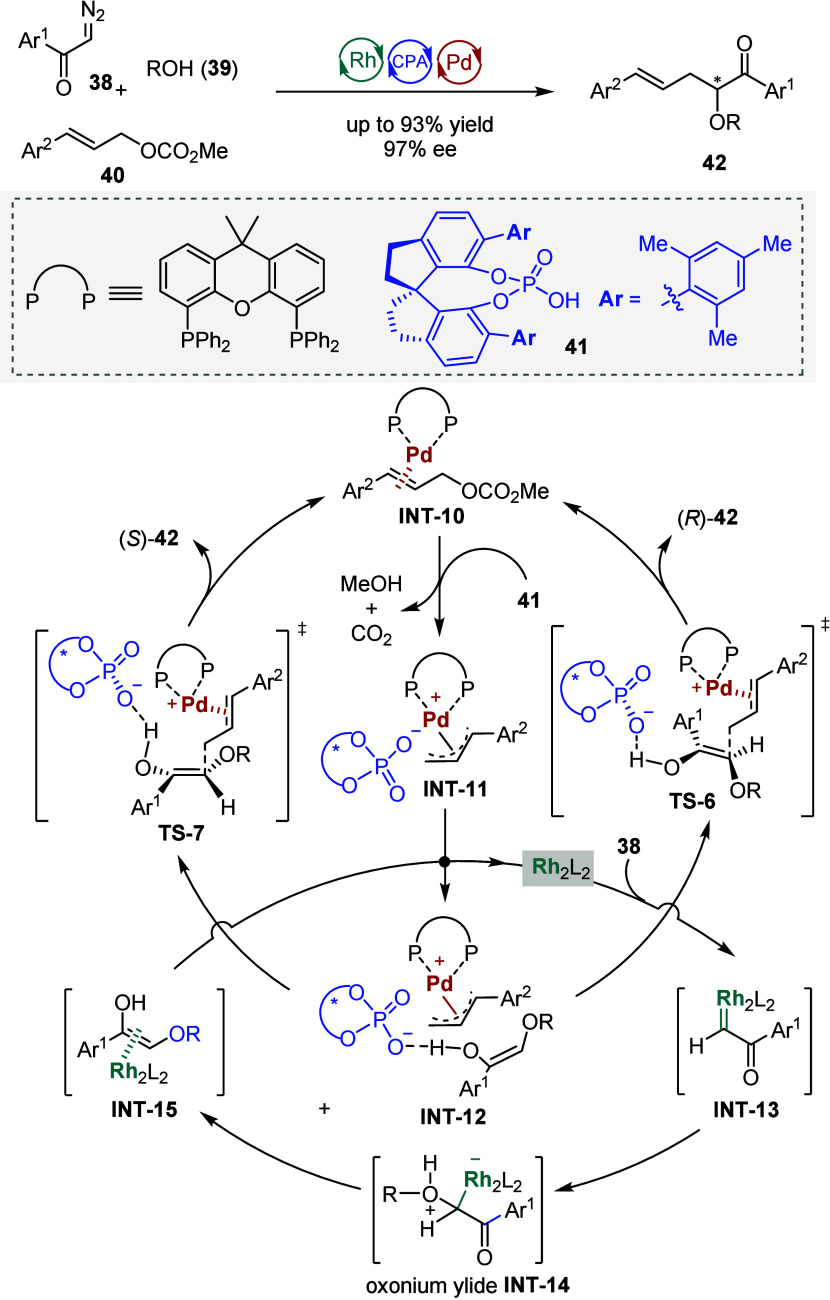
Triple Rh­(II)-Chiral Phosphoric Acid-Pd(0) Catalysis for Substitution-type Trapping of Onium Ylides

The consecutive construction of chemical bonds in multiple-component reactions (MCRs) can significantly reduce material, labor, and time costs, making them highly appealing for industrial applications. A notable example is the transformation of syngas and its subsequent derivatization. In 2018, Han and Gong reported a quadruple catalysis system consisting of an achiral Rh­(I) complex, amine, Pd(0) complex, and a CPA catalyst **47** (Scheme 6).^114^ Starting from syngas, the in situ-generated rhodium hydride complex coordinates with styrene derivatives **43** and then undergoes a migratory insertion reaction to form alkyl rhodium species **INT-18**. Ligand exchange of **INT-18** with CO affords **INT-19**, which undergoes sequential carbonylation, oxidative addition with H_2_, and reductive elimination to release branched aldehydes **48**. HOMO activation of **48** by the quaternary amine **45** gives an enamine intermediate **INT-22**, which then participates in an enantioselective allylic alkylation reaction^115^ with a π-allyl-palladium chiral phosphate **INT-23** generated from allylic alcohol **44**, Pd(0) complex, and CPA **46** (Scheme 6). The pressure of syngas played a crucial role in this MCR, as excess CO could poison the Pd(0) complex, halting the entire catalytic cycle, while a low syngas pressure typically led to low-yielding hydroformylation reactions. Consequently, 1 bar of syngas was employed to optimize the reactivity and leverage the competing effects.

**Scheme 6:**
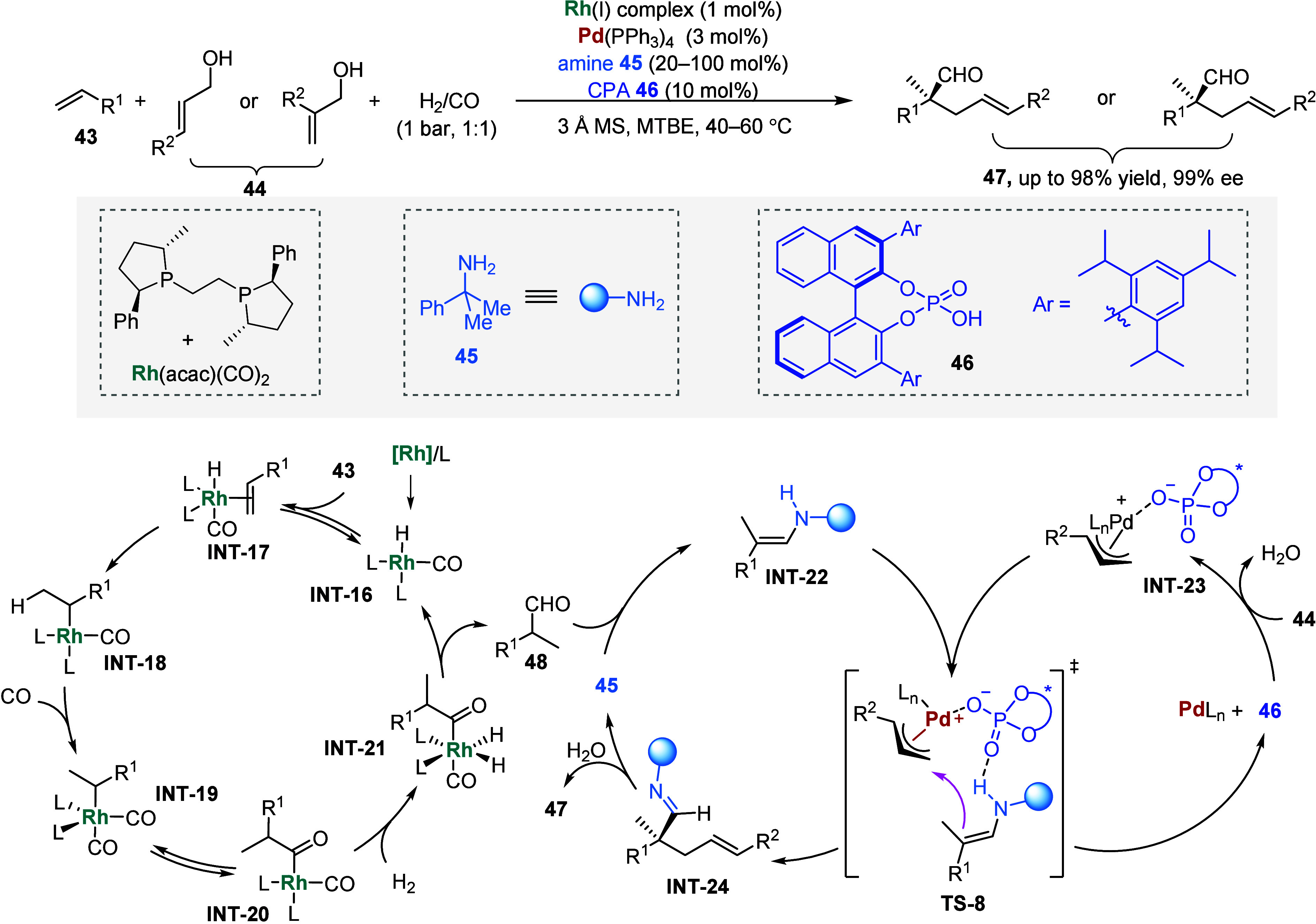
α-Quaternary Chiral Aldehydes from Styrenes, Syngas, and Allylic Alcohols via Quadruple Relay Catalysis

### 2.4 External Stimuli-Endowed Organo-Metal Combined Catalysis

In contrast to ionic intermediates, harnessing highly reactive radical species for the synthesis of enantioenriched molecules still remains a significant challenge. A novel redox strategy for organic synthesis through external stimuli, such as light and electricity, has seen an explosive development in recent years.^116^ Particularly, photoredox catalysis, utilizing molecules capable of harvesting visible light, has emerged as a mild and versatile approach to tame the generation and transformation of radicals.^117,118^ The recent integration of photoredox catalysis with transition metal catalysis, termed metallaphotoredox catalysis,^119,120^ has further expanded the potential for bond formation. The core processes of photoredox catalysis, including single-electron and energy transfer, can be triggered by either transition-metal-based photocatalysts or purely organic dyes, a topic that has been extensively reviewed. In this section, we focus on the emerging field of multiple asymmetric catalysis, which combines photoredox, organocatalysis, and transition-metal catalysis.

Nature has inspired higher-order chemical processes to create complex molecules, including multiple component reactions with high atom and step economy, as well as multiple catalysis capable of forging otherwise inaccessible chemical bonds.

In 2022, Luo and colleagues utilized photoredox-catalyzed single-electron oxidation of a chiral enamine intermediate^121^ to enable an asymmetric C–H dehydrogenative allylic alkylation reaction.^122^ As shown in Scheme 7, the oxidative quenching of an Ir-based photocatalyst Ir­(ppy)_2_(dtbbpy)­PF_6_ **51** in its excited state by cobaloxime **53** generated an Ir­(IV) species and a Co­(II)-metalloradical. The oxidation of the in situ generated chiral enamine **INT-25** by the Ir­(IV) complex, followed by deprotonation, yields an α-imino radical **INT-26**. The cooperative addition of this radical and the Co­(II)-metalloradical to alkene **50** produced alkyl Co­(III) intermediate **INT-27**, which undergoes photochemical dehydrogenation to afford the allylation product **INT-28** and a Co­(III)–H species. Subsequent hydrolysis of the imine motif in **INT-28** affords the final product **54**, and heterolytic C–H cleavage by a proton regenerates the chiral amine **52** and the Co­(III) catalyst. Control experiments indicated that no product was formed in the dark, and elevated temperature (from −20 °C to room temperature) resulted in improved reactivity but with reduced stereoselectivity. This work has exemplified a triple-catalysis strategy to address challenges in bond formation using planar Co­(II)-metalloradicals.

**Scheme 7:**
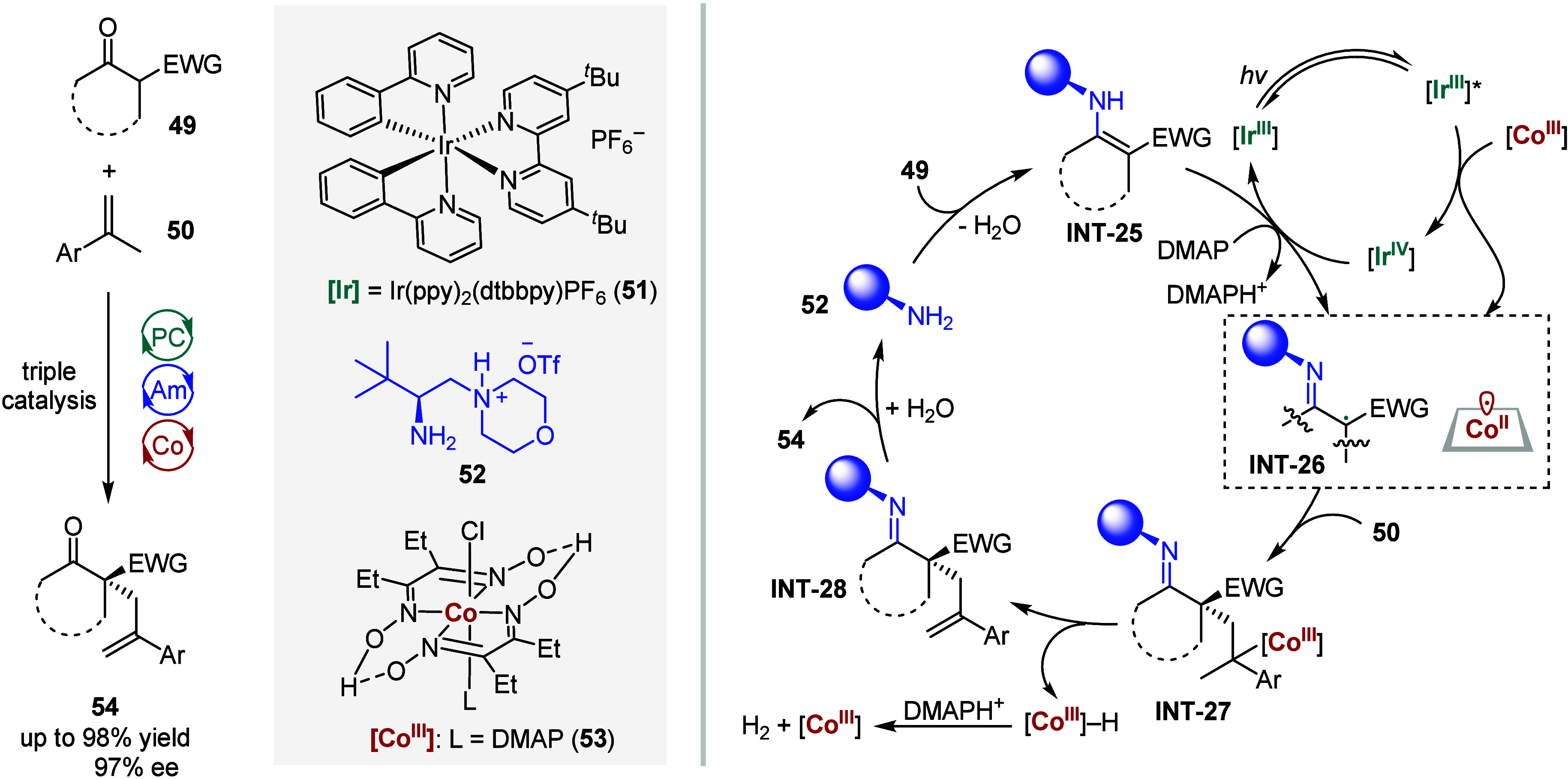
Ternary Visible-Light Photoredox-Cobalt-Chiral Primary Amine Catalysis Enabled an Asymmetric C–H Dehydrogenative Allylic Alkylation Reaction

Recently, Yang et al. demonstrated a triple photoredox-Fe-chiral primary amine catalysis,^123^ overcoming the challenges associated with chiral Fe­(III)-metalloradical catalysis in accessing quaternary stereocenters through alkyl–alkyl cross-coupling. As shown in Scheme 8, single-electron oxidation of the in situ generated chiral enamine **INT-29** by a highly oxidizing excited-state Ir-based photocatalyst Ir­[dF­(CF_3_)­ppy]_2_(dtbbpy) PF_6_ **57**, followed by deprotonation, produces an α-imino radical **INT-30**. The reduced Ir­(II) complex then donates an electron to the redox-active NHPI ester **56**, initiating a decarboxylation process that generates a primary alkyl radical **INT-31**. This radical **INT-31** is captured by the Fe­(II) complex **59**, forming an alkyl-Fe­(III) species **INT-32**, which subsequently reacts with the α-imino radical **INT-30** via an outer-sphere radical rebound mechanism. Finally, hydrolysis of the chiral imine **INT-33** yields the alkylated product **60** in good yield and excellent enantioselectivity while regenerating the chiral primary amine catalyst.

**Scheme 8:**
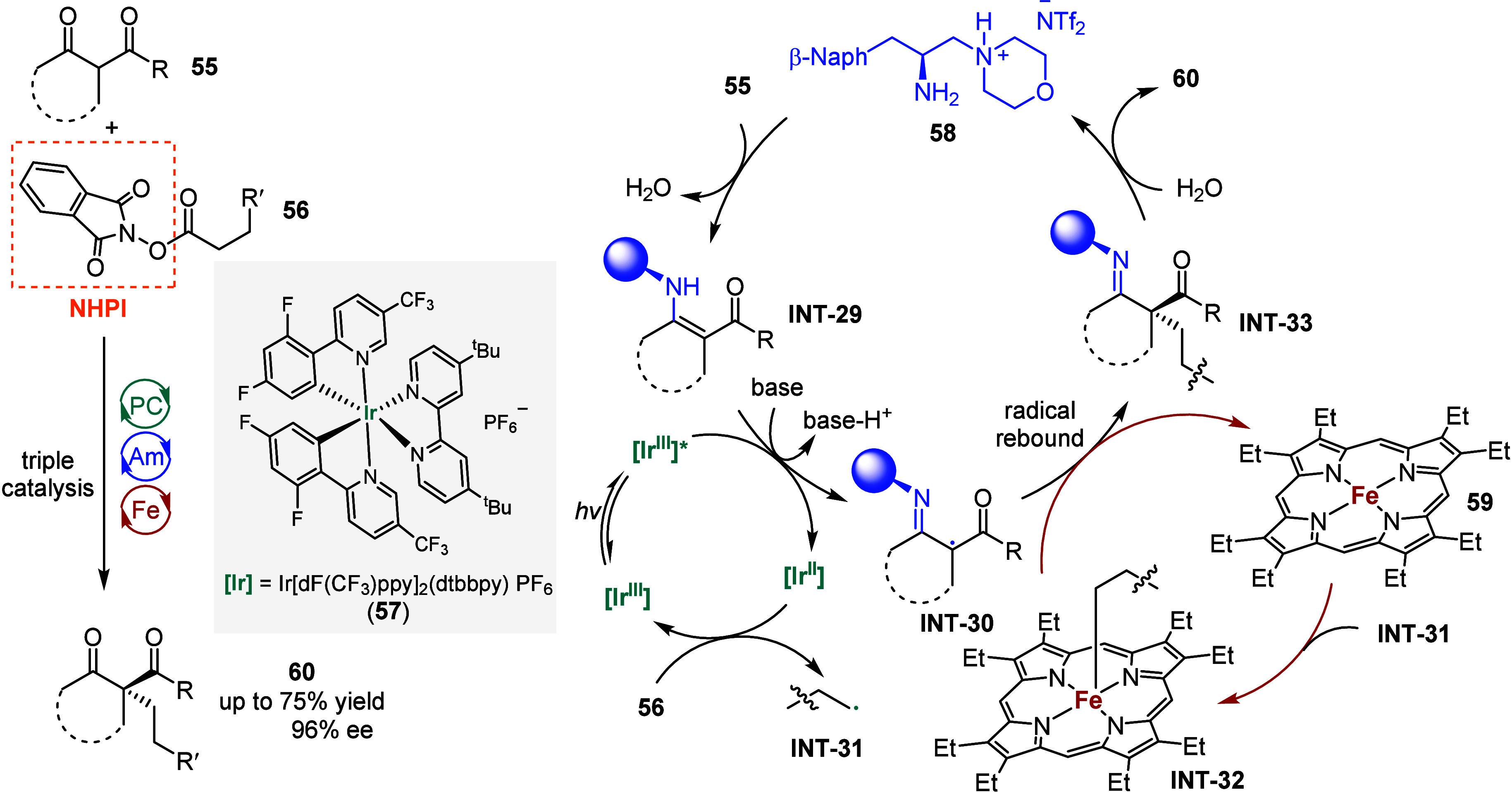
Triple Photoredox-Fe-Chiral Primary Amine Catalysis Enabled Enantioselective Construction of Quaternary Stereocenters

## 3 Conclusion and Perspectives

In conclusion, we have provided a brief overview of the early development of asymmetric organo-metal combined catalysis and outlined the general activation modes employed in numerous bond-forming processes. These processes, often enabled by the use of chiral organocatalysts, exhibit a high degree of stereocontrol. While the field has evolved from dual to multiple catalysis, the most exciting and practical progress lies in the discovery of unprecedented catalyst scaffolds. These scaffolds enable otherwise inaccessible transformations and provide unparalleled capacity for combined catalysis in various subfields of synthetic chemistry.

With that in mind, we offer our perspective on the future opportunities to pursue in the field of asymmetric organo-metal combined catalysis:

(1) **New catalyst scaffold:** Beyond the rational design of covalently bonded ligands, recent work by Ooi, Toste, and Phipps has highlighted the unique and flexible role of ionic interactions in tuning both the steric and electronic properties of metal centers. Remarkably, Jacobsen and colleagues^124^ employed a chiral bis-thiourea hydrogen-bonding donor to bind the anion of a cationic transition-metal complex. This cooperative anion-binding approach not only accelerated the rate of a Ru-catalyzed intramolecular propargylic substitution reaction but also induced high enantioselectivity (up to 99% *ee*). This example provides a solution to the challenge of poor stereochemical communication in traditional chiral ligand–transition metal catalysis.
(2) **Heterogeneous catalysis:** The development of well-defined heterogeneous metal catalysts,^125^ especially those utilizing surface modification techniques, and supported chiral organocatalyst,^126^ offer exciting opportunities to unlock new chemical processes with unprecedented reactivity, selectivity, and sustainability. For example, Wang et al.^127^ has reviewed the influence of ligands on single-atom catalysts (SACs)^128^ and their growing application in organic synthesis, although the asymmetric version of single-atom catalysis remains underexplored. Recently, Xiao and Ge^129^ achieved a cascade Suzuki coupling and asymmetric kinetic resolution reaction by anchoring a lipase to the Pd-based SAC. This work has the potential to inspire broader applications of heterogeneous catalysis in conjunction with enzymatic or organocatalysis in asymmetric synthesis. Despite notable advances, future development of heterogeneous asymmetric organo-metal combined catalysis may consider the synergistic effect of supporting materials, facile recovery of catalysts, reproductivity, scalability, etc.
(3) **Electrochemical catalysis:** Electrochemistry, as a rapidly advancing “green” technology, offers the potential to significantly expand the capabilities of traditional metal or organocatalysis.^130–132^ It facilitates the straightforward generation of reactive intermediates and/or supports the regeneration of redox metal catalysts. Additionally, the combination of photocatalysis and electrocatalysis—known as photoelectrocatalysis or electrophotocatalysis^133–135^ —has found increasing application in asymmetric synthesis.^136^ We believe that integrating electrochemical or photoelectrochemical regulation with asymmetric organo-metal combined catalysis will further enhance its efficiency and open new avenues for synthetic chemistry.
(4) **Macromolecular synthesis:** The stereoregularity of polymers plays a profound role in determining their material properties. Achieving stereoselective polymerization that controls potentially hundreds of consecutive stereocenters still remains a grand challenge.^137^ Inspired by asymmetric ion-pairing catalysis, Leibfarth et al.^138^ combined a chiral phosphoric acid with TiCl_4_ for the highly stereoselective cationic polymerization of vinyl ethers, yielding isotactic poly­(vinyl ethers). This work serves as a reminder of how catalysis can reshape fundamental polymerization concepts and drive advancements in macromolecular synthesis.^139^ Future efforts may be directed to the discovery of novel catalyst combination that can polymerize a broad scope of vinyl monomers, as well as the development of industrial-relevant large-scale synthetic technologies.
